# The Contribution of Mycological Tourism to Well-Being, the Economy and Sustainable Development

**DOI:** 10.3390/ijerph192417027

**Published:** 2022-12-18

**Authors:** Pablo Suazo, Alba Viana-Lora

**Affiliations:** 1 Faculty of Administration and Economics, Tarapacá University, Iquique 1100000, Chile; 2 Department of Geography, Rovira i Virgili University, 43480 Vila-seca, Spain

**Keywords:** mycotourism, mycological tourism, mushroom tourism, well-being, sustainable rural development

## Abstract

This article analyses the scientific production related to tourism and mushrooms. The method used was a bibliometric analysis and a systematic literature review. The main results show that it is a recent area of study that predominates in Spain but that will expand and gain relevance over time. The thematic analysis has made it possible to structure the information according to the economic contribution of this tourist niche, the well-being it brings to residents and tourists, the importance of a sustainable development of the activity, and the promotion and marketing of this new tourism. Supporting mycological tourism will help the development of rural areas and bring physical, mental, social, educational, and nutritional benefits to residents and tourists. This study has allowed us to develop a future research agenda, highlighting the importance of further research to harness the benefits of mycological tourism while at the same time transferring that knowledge to stakeholders, which will be necessary.

## 1. Introduction

The development of tourism experiences provides us with a considerable source of research because the structure of the offer is linked, to a greater or lesser degree, to the immersion in spatial contexts, to the emotions and satisfaction of tourists. In terms of accession, we can establish very simply that mycological tourism is a form of living experiences associated with fungi, which is conceptually linked to other broader definitions, such as: rural tourism, ecotourism, gastronomic, scientific, cultural, wellness or health tourism [1,2,3]. Therefore, the possible definitions of mycological tourism move between different dimensions and classifications that represent perspectives of analysis and thematic orientations that each researcher considers relevant according to their area of interest and study. Perhaps the most consistent definition determines that mycological tourism is a specialised branch of the offer provided by ecotourism, which is made up of collecting, gastronomy, and a series of other socio-cultural activities demanded by tourists and which, as a whole or in a particular way, manages to generate profitability [2]. However, beyond definitions, it is necessary to reflect on certain dimensions where mycological tourism can extract the essence of its origin, performance, and perspective. Fungi are fruiting bodies found in or under the soil and may or may not be edible [4]. They are integrated into different ecosystems and contribute to their balance, their main characteristic being that they contribute to the decomposition of organic materials through diverse functions and biological interactions that favour mediation between different organisms and ecosystems [5]. Fungi reproduce through spores, which are microscopic cells that reproduce among themselves; their germination produces the mycelium, which we can consider the “root” of the mushroom [6,7]. Fungi are second only to insects as organisms present in nature [8]. There is a diversity of types of fungi, both unicellular and multicellular, different shapes and sizes with dissimilar colours and odours that achieve an intrinsic richness from their usefulness for the environment, and therefore for humans, to their aesthetic beauty linked to the landscapes where they reside [4]. Mushrooms are also associated with socio-cultural dimensions in terms of rural development through the collection and gastronomic use of mushrooms [9]. From this background, mycological tourism has an exceptional basis when it comes to the creation of experiences and products from which sustainability in the conservation and protection of mushrooms is derived in the first place [10]. The ecological importance is another reason why mycological tourism, together with other types of nature tourism and ecotourism, can become a promoter for the protection of these organisms [11]. Obviously, it is necessary that mycological tourism is developed through environmentally responsible practises and programmes that promote awareness of the importance and significance of fungi for the environment [12]. The scientific perspective provides mycological tourism to be involved with scientific dissemination and the study of fungi in a broad sense, especially to know the classification and taxonomy considering that there are approximately 100,000 species of fungi [13]. It is also relevant to understand the composition of their structures, evolution, and adaptive processes that fungi develop in different natural environments. These elements are constitutive to generate experiences for tourists interested in the scientific knowledge of fungi, where mycological tourism can respond by creating specialised offers. In relation to the above, it is known that the healing properties of mushrooms are a contribution to the life sciences [14]. In many parts of the world and in their respective cultures, mushrooms have been consumed and applied in treatments for different health problems [15,16], hence mycological tourism assumes the creation of healing experiences with medicinal therapies along the lines of health tourism, providing benefits and wellbeing to those who resort to this type of offer. In terms of wellbeing, wild mushroom picking is an activity that is carried out in various parts of the world, for example, in Europe, mushroom picking is a very important recreational forestry activity [17]. Mushrooms produce health benefits in both the psychological and physical domains, which are linked to their relationship with the environment, and even mushrooms possess a magical attraction [18].

Therefore, mushroom picking as a recreational activity and knowledge and contact with wild spaces are immersive experiences for tourists, who value disconnection, learning, and the promotion of awareness of nature. In this perspective, mycological tourism should take advantage of the opportunities offered by the need to visit and inhabit rural or less intervened spaces where people of different ages and conditions integrate recreation, rest, knowledge, disconnection, and mindfulness [19]. This is undoubtedly a rewarding exercise, especially if it has been carried out after the confinements caused by COVID-19 [20]. Finally, gastronomy is where the development of mycological tourism is best embodied, as it harmonises gathering, knowledge of mushrooms, their nutritional properties, the rescue of culinary heritage, rural culture, innovation, and the dissemination of products. Currently, edible mushrooms enjoy wide popularity, however, it is necessary to take precautions to reduce threats to their conservation [21]. In this context, mushrooms are a bio-cultural resource rooted in local culinary forms that are manifested in tourism routes, tastings, local and Indigenous markets, festivals, tastings, and, most identifiably, the gastronomic experience in restaurants [22]. As we have expressed, mycological tourism is combined with different dimensions that can act separately or in combination when it comes to establishing an offer. Following this line of argument, this paper aims to respond to the need to describe the dimensions and research topics addressed in mycological tourism, as well as to develop a future research agenda considering the possible difficulties regarding the conservation of the resource.

The rest of this paper is structured as follows: Section 2 describes the method used in the article, which consists of a bibliometric analysis and a systematic literature review. Section 3 presents the results of the study, structured according to the most relevant themes for the research. Section 4 discusses the results of the study. Section 5 presents an agenda for future research. Finally, Section 6 concludes the analysis.

## 2. Materials and Methods

The method used in this article combines a bibliometric analysis and a systematic literature review on tourism and mycology. On the one hand, bibliometric analysis is used to explore and map the scientific knowledge of a specific field [23]. Bibliometrics began in the 1950s, although it has been increasing over the years thanks to advances in analysis techniques and the growth of scientific research [23,24]. The development of databases such as the Web of Science or specialised software such as VOSviewer has facilitated the production of these reports [25,26,27]. On the other hand, the systematic literature review makes it possible to describe the state of the literature in a thematic area, test a specific hypothesis, extend the literature through new constructs, or compare the literature through established criteria [28]. It is a procedure for a comprehensive evidence-based discussion [29]. In addition, the transparency of both procedures enables replication in other fields of study. The database used to extract the information was the Web of Science (WOS), which is widely used in bibliometric analyses and systematic literature reviews [30]. Its use is due to the ease of access to publications from all fields of knowledge and its high-quality index [26]. Its quality criteria are stricter than those in other databases [31]. Its design allows access to content in an organised and structured way [32], performing specific searches with a high level of thoroughness [30,33]. The use of a single database allows us to standardise the analysis process [34].

**Figure 1:**
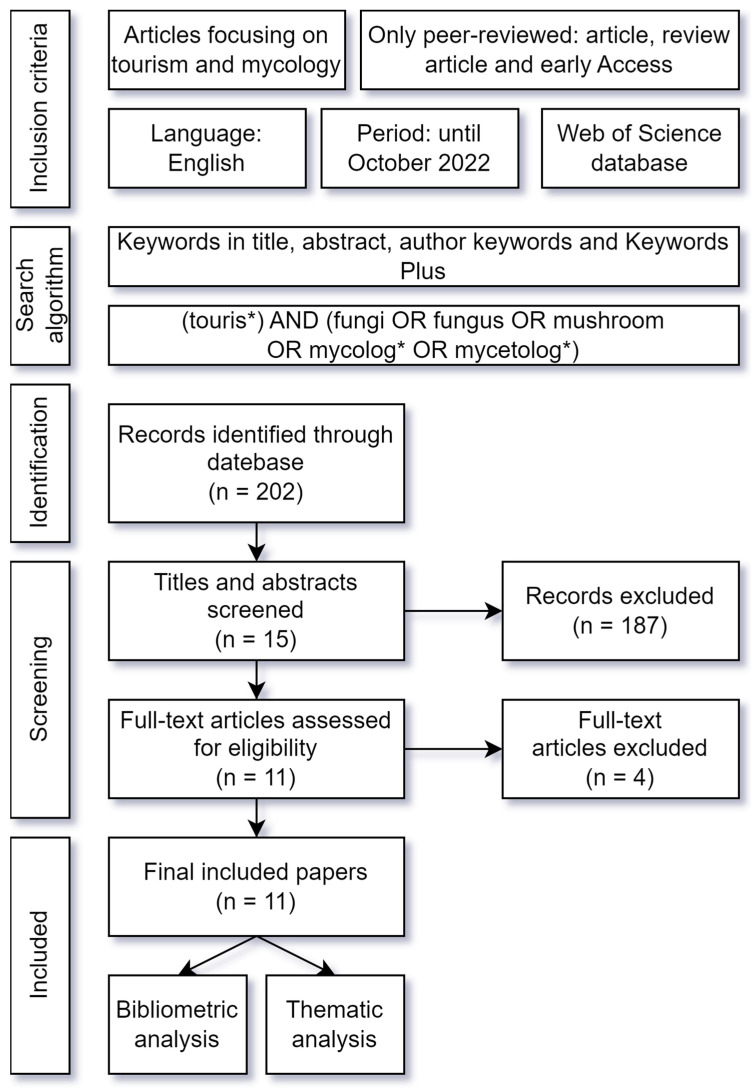
Review protocol. Own elaboration. * The asterisk has been used in the search algorithm to capture all derived words.

For the process of downloading documents and subsequent analysis, a review protocol was drawn up that included the inclusion criteria, the search strategy, and the phases of analysis (see Figure 1).

The WOS database was searched following the inclusion criteria and the search algorithm in Figure 1, which allowed the selection of documents that, in addition to including the words derived from “touris*” contained any of these five terms: “fungi”, “fungus”, “mushroom”, “mycolog*”, and “mycetolog”. We did not filter by subject category to reach journals beyond the area of tourism. To select the highest quality publications, the document type was selected as “article”, “review article”, and “early Access”. The language selected for the publications was English, and this study could be replicated in another language to compare the results. The procedure was carried out at the end of October 2022 and resulted in 202 articles. This was followed by a filtering process in which the authors read the title and abstract of each article to exclude papers that did not focus on tourism and mycology, with 15 articles continuing to the next screening stage. The next screening consisted of reading each article in full, excluding 4 articles, and selecting 11 articles for the next phase of analysis.

The VOSviewer software is used to graphically represent the results of the bibliometric analysis. It is a very useful tool whose operation is based on the visualisation of similarities, allowing easy mapping of information [27]. VOSviewer is widely used in bibliometric analysis, as it allows a wide range of networks composed of publications, authors, journals, organisations, or countries to be analysed [35]. In addition, a thematic analysis is performed on the selected documents to identify the main themes and main sub-themes [36], the results of which are presented in the following section.

## 3. Results

There were 11 publications on tourism and mycology analysed in this study. The scarce scientific production is due to the fact that it is a new subject, with the first publication dating from 2012 and an increase in interest from 2021 onward, when three articles were published. The scientific interest in mycological tourism may be related to the increase in the practise of this type of tourism, probably influenced by the changes in trends after the COVID-19 pandemic focused on the search for activities related to nature and wellbeing [20]. It is a good time to analyse the scientific production and guide the research towards the most relevant topics. This section has been divided to highlight the results of the bibliometric analysis on the one hand and the systematic literature review on the other.

### 3.1. Bibliometric Analysis

The bibliometric analysis carried out allowed us to detect the keywords, authors, countries, the citations, journals, and references, which appear in the scientific production of tourism and mycology.

#### 3.1.1. Keywords

**Figure 2:**
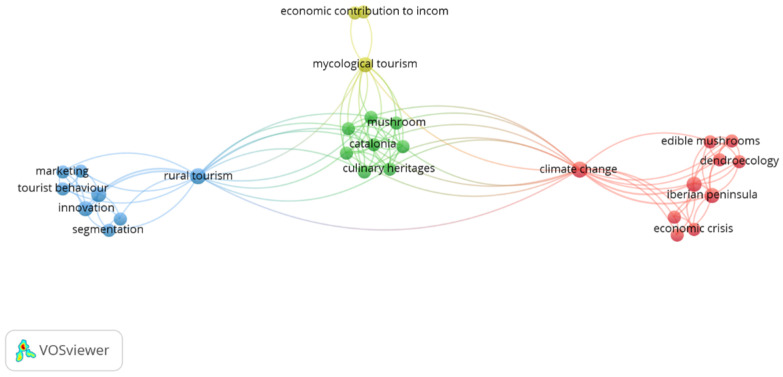
Keywords. Own elaboration.

The keywords used by the authors total 52. We used the VOSviewer software, which detected the connection of 29 of these keywords, which are represented in Figure 2. The words that appear repeatedly in several articles are “climate change”, “rural tourism”, “iberian peninsula”, “innovation”, “non-wood forest products” the derivatives of mushroom tourism, “mycological tourism”, and “mycotourism”. The concepts were organised into four main clusters according to their relationships. The repetition of these keywords suggests a strong relationship between mycological tourism and sustainable rural development due to its link with the natural environment. In addition, the keyword “iberian peninsula” indicates the area of study in which most scientific production has been developed.

#### 3.1.2. Authors and Countries

**Figure 3:**
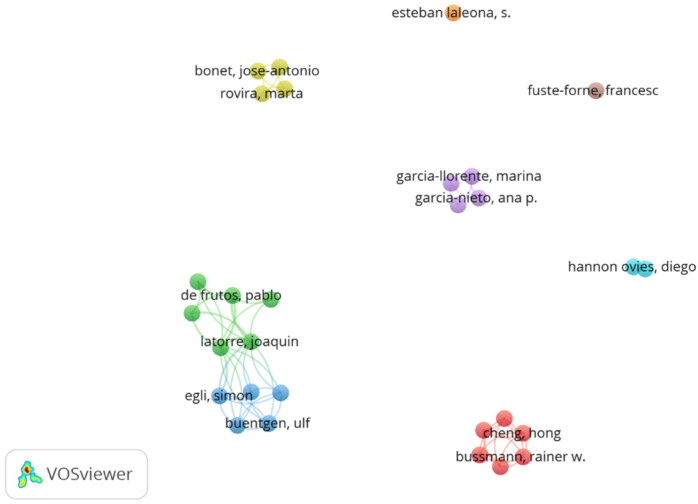
Authors. Own elaboration.

There were 29 authors who published, with an average of 4 authors per publication. These authors were mapped with VOSviewer in Figure 3. Of the 11 articles, 6 share an author, so this field of research currently has a network of authors who are related to each other. In addition, most of the authors are linked to Spanish institutions, so that nine of the publications have an area of study in Spain. The other two publications are from China and Mexico.

#### 3.1.3. Citations, Journals and References

The analysed articles were cited 336 times. The article “Mapping forest ecosystem services: From providing units to beneficiaries” with 203 citations was the most cited, followed by “Drought-induced changes in the phenology, productivity and diversity of Spanish fungi” and “Socio-economic, scientific, and political benefits of mycotourism”, with 38 and 34 citations, respectively. A query was made on the same date in Google Scholar, and the citations of these three articles increased respectively by 314, 48, and 46 citations. The publications are from 11 different journals, so each of the articles was published in a different journal. Four of the journals are ranked in the tourism branch, and nine of them are positioned as first quartile in the Scimago Journal & Country Rank (SJR). A summary of this information can be found in Table 1.

To complete the bibliometric analysis, VOSviewer was used to analyse the references in each publication. It was found that the publications cited a total of 765 documents, 47 of which were repeated in several articles. The journals cited amounted to 518, and 4 journals were found to appear in all documents. In addition, VOSviewer allows the first author of each reference to be selected, resulting in 656 authors cited in the 11 publications.

### 3.2. Thematic Analysis

Wild mushrooms have been consumed throughout human history since their appearance in the Palaeolithic era [39]. Their study and usefulness to humans falls within the branch of biology called mycology [43] and the relationship between these fungi and people is found within ethnomycology [21]. Mushrooms are considered a non-timber forest product (NTFP), “products of biological origin other than wood derived from forests, other wooded land and trees outside forests” [38,43,44]. The collection of wild mushrooms is an important activity for many countries. However, tourism promoting this activity is relatively new [40,41,42]. “Mushroom tourism”, “mycotourism”, or “mycological tourism” has become a specific product considered unique and innovative due to its characteristics [40,42]. It has been analysed in the following sections according to the most relevant issues for the research.

#### 3.2.1. Economic Benefits of Mycotourism

The mushroom world has an impact on the economy. In countries such as China, the edible mushroom industry has become an important economic factor, being the leading country in production [21]. In turn, the tourism sector has developed a niche tourism related to mushrooms, which is a great source of income through the consumption of this product or the development of activities, events, festivals, and rituals [38,39,45]. Examples are the “Mushroom Food Culture Festival” of Nanhua County (China) [21], the “International Wild Fungi Festival” in Piedra Canteada (Mexico), the “Christchurch Mushroom Festival” (New Zealand) [43], the “Gastronomic Mushroom Festival” in Alta Ribagorça (Spain) [39] or the ancient mushroom rituals in Oaxaca (Mexico) [45].

Mushroom tourism contributes favourably to the economies of Spanish regions such as Catalonia, with an economic impact of 800,000 euros per year [39], or Castilla y León, with an average annual expenditure associated with mycological tourists of 4.5 million euros and an annual job creation of 46 jobs on average [40]. It has positioned itself as a tourist activity that enhances the value of villages and rural areas, diversifying the offer, favouring economic development, and restoring their social fabric [38,39,42,43,45].

#### 3.2.2. Contribution of Mycological Tourism to Well-Being

Mushroom tourism provides benefits other than economic [1], such as access to products provided by nature, social interaction, promotion of culture, and physical, emotional, and spiritual well-being [39]. It is considered an ecosystem service because of its contributions to humans [37]. The tourism experience provided by mycological tourism can improve health status. On the one hand, we find that the practise of this tourism is linked to the development of physical activity in the natural environment, and it has been proven that sporting activities improve health and well-being [42]. On the other hand, mushrooms are considered a healthy wild food that is beneficial to health [39] due to their high content of bioactives and vitamins [42]. Furthermore, the satisfaction of self-improvement and the emotions of spirituality and inner well-being influence health [42]. In addition, mushroom picking has been considered a way of life and part of the local culture of rural populations, so mycological tourism promotes and safeguards the traditions of the villages and transmits the personal feeling towards the place that its population has [39]. The Mexican community of Oaxaca, known for its “magic mushrooms”, has a tourism promotion strategy for the consumption of these mushrooms through rituals or *veladas*, claiming the therapeutic potential of these psilocybin mushrooms [45]. For them, these rituals present a form of healing and increase the wellbeing of the participants. [45] We can therefore say that this tourist activity improves the quality of life of society as a whole [42].

#### 3.2.3. Towards a Sustainable Development of Mushroom Tourism

Mushroom picking is an activity carried out in nature and therefore strongly related to the environment [37]. The uniqueness surrounding the formation of mushrooms highlight the need to preserve the land and its ecosystem [39]. Therefore, the focus should be on related activities, such as mycological tourism. Mycological tourism is an opportunity to value and recognise the importance of environmental conservation [42]. However, knowledge related to this practise is increasingly limited, possibly due to the rural exodus and loss of contact with natural resources [39]. There is a need to raise awareness and educate this type of tourist by disseminating traditional and Indigenous harvesting practises or transferring scientific knowledge on the subject [1,21]. Good harvesting practises such as, for example, leaving part of the mushroom production to regenerate [43], not harvesting immature specimens, or always carrying baskets with grids that favour mushroom sporulation in the field while the activity is being carried out are some of them. Mycological tourism can use as an example ecotourism practises that serve to minimise the impact of the activity on the environment and to be oriented towards responsible tourism [1,43]. In fact, mycotourism is considered a sustainable tourism activity [43]. Latorre et al. [43] propose reinvesting the profits generated by the activity in conserving the resource. However, there are external factors that have a negative impact on the development of mycological tourism, such as climate change. Climate change causes droughts, sudden changes in temperature and a decrease in the amount of forests [38], which alters the mushroom ecosystem and has a significant effect on their development [39]. It has been shown that climate-influenced mushrooms are increasingly late-fruiting, with fewer mushrooms and a reduction in size and species [38]. This type of tourism requires precise planning due to the importance of the natural environment for humans [39]. It is important to know that mushrooms are the property of the owner of the land on which they are found [43]. The management of public areas where mushroom tourism is practised has been one of the measures carried out by managers, as in the case of Castilla y León, which has designed an application to reserve and control the collection of mushrooms in regulated forests [1]. It is a measure that can help sustainability in the long term [41], but it must be supported by national policies that control the activity [43]. The activity is expected to grow, so regulation [41] is essential to ensure its protection [43] and to achieve sustainable tourism development.

#### 3.2.4. The Importance of a Correct Promotion of Mycotourism

Mycological tourism is considered a new tourism trend [39], opening the way for a wide range of promotion and marketing. Destinations can benefit from local mushrooms and fungi and increase their tourist attractiveness [44]. The creation and consolidation of recognisable tourism brands in the harvesting areas that allow their identification will be a strategy to be carried out [21]. However, in order to promote these areas for tourism, a prior analysis of the mycological tourist profile, their main motivations and behaviours is needed [43]. Latorre et al. [43] made an approximation of the profile of the mycological tourist in Castilla y León and determined that they are mainly women (55.1%) who are between 36 and 60 years old (62%) with university studies (57.6%) and a family income of between 601 and 1800 euros (53.9%). This study highlights that overnight tourists account for 39.8% of the total with an average stay of 4 days and a main choice of private accommodation (13.9%) [43]. De Frutos Madrazo, Martínez-Peña, and Esteban [40] used the same study area to estimate average annual overnight stays, expenditures, and employment. Despite sharing space with other types of tourism, such as rural, gastronomic, cultural, sports, health, ecotourism, or wine tourism [42], their motivations vary and require specific studies on the subject. The fact of going to a place to pick mushrooms is considered a motivation to make the trip [42,43], but mycotourists will choose their destination based on the production of the area, the existing infrastructure, and the cultural events related to the activity [40,42]. Accessibility will also be a determining factor [43]. Mycological tourism can be offered through individual activities or packages containing mycological routes or days, product sales, visits to specialised mycological centres, and cultural and gastronomic events [40,42]. The planning of these activities is complex due to their seasonality, i.e., the seasonal availability of the product, as it depends on weather factors, such as rainfall, temperature, or wind, but at the same time, this seasonality makes the activity unique as it offers a different experience each time it is carried out [39].

## 4. Discussion

The increase in mushroom picking activities opens up a niche market in tourism, mycological tourism, which favours rural development. Residents of rural areas will be able to improve their living conditions through the benefits generated by mycological activities. This activity fosters economic growth. It has been shown that mycological tourism has the potential to create quality jobs and increase tourism expenditure in the region [40]. Therefore, different entities of a destination will benefit economically, such as accommodation companies, tourist guides, or restaurants in the area. Mycological tourism allows the transmission of local tradition and the enhancement of local culture, expanding knowledge and skills in mushroom gathering.

This activity brings numerous benefits related to well-being. The practise of this activity improves the physical condition of the tourist, at the same time as it brings mental benefits related to inner well-being and the development of the senses of sight, smell, touch, and taste. It sharpens the wits and enhances orientation and the sense of space and time. There are also social benefits to doing the activity in company and meeting new people, socialising helps keep your brain healthy and increases self-esteem [46]. The educational benefits of learning about the environment and the world of mushrooms produce personal satisfaction and allow you to connect with nature. In addition, as discussed above, mushroom consumption provides dietary benefits.

Climate change is a relevant and important issue for the planet. As evidenced in this article, it has an important effect on mycological tourism. Mushrooms require specific climatic conditions. Further progress must be made in protecting the environment in order to curb the effects of climate change, which is why research plays an important role.

Mycological tourism, like any other niche tourism, requires specific promotion and marketing that is adapted to demand, but there is a lack of research on the subject. This article has detected the few publications on the subject and the concentration of studies in specific areas of Spain, so the need to continue advancing in this line of research is highlighted, and a future research agenda is developed in the following section.

## 5. Future Research Agenda on Mycological Tourism

This study has shown that there is a significant lack of research on mycological tourism. However, the articles analysed coincide in the multitude of benefits that this type of tourism brings to the population. The increase in scientific interest in this subject took place in 2021, so an increase in these types of publications is expected over the next decade. We therefore consider that this research should be aimed at filling the gap detected in the following four lines of research.

Firstly, studies on mycological tourism should make planning proposals. On the one hand, the increase in this type of tourism can lead to the overexploitation of certain forest areas, so research will need to focus on the development of new regulations or policies to control the activity and guarantee the sustainability of the ecosystem. There is potential for other research to address the impacts on local economies as an endogenous development factor and to be linked to analyses that quantitatively and qualitatively determine the effects of investment, the price-quality ratio, or the participation of women and young people in the employment generated by mycological tourism. In turn, research can provide information on the agro-productive externalities related to the presence of mycological tourism and its contribution as a factor of territorial balance between urban and rural areas. Therefore, more research is needed to ensure that mycological tourism contributes to the quality of life of residents and demonstrates the benefits for rural populations. On the other hand, the importance of accommodation and catering infrastructure for mycological tourists in their choice of destination has been explained, and studies focused on the design and creation of infrastructure to support the activity are needed, since a destination that plans and invests in infrastructure will have a greater chance of benefiting from mycological tourism. 

Secondly, a new line of research is opening up on the awareness and education of the mycological tourist. On the one hand, mycological tourism is carried out in the natural environment, so its conservation is key to the continued existence of the activity. The tourist must be educated with good practises based on responsible tourism, as well as correcting and redirecting behaviour that is not respectful of the environment. In this direction, it is also necessary to know how mycological tourism contributes to improving the social function of ecosystems based on the coexistence between tourists and local inhabitants, with reference to the individual and collective appreciations that are produced in the interaction that the tourist activity entails. Another pending area of research is related to the link between mycological science and the contribution that mycotourism can make to its dissemination. On the other hand, the mycological tourist must have knowledge about mushroom picking since the consumption of inedible mushrooms brings danger to this tourist activity. 

Thirdly, we have detected studies that analyse mycotourism only in a specific area, Castilla y León (Spain), which opens up a range of analyses of the profile of this type of tourist in the rest of the world’s mushroom gathering areas. Along these lines, research challenges also arise in terms of identifying the motivations and emotions generated by experiences in mycological tourism in order to obtain information to optimise their performance in terms of the construction of supply and knowledge of possible demands. This aims at research to determine the preferences on which tourists focus when deciding which mycological tourism offer to take. In addition, it is necessary to advance in research that illustrates the value chain and helps to detail in precise ways the commercial organisation of mycological tourism.

Finally, given the importance of this tourism for health and the new behavioural patterns after COVID-19, in which activities related to wellbeing are sought, mycological tourism becomes an alternative that requires research that presents information on how the offers are adapting to post-pandemic demands and how visitors are mobilised in search of mycological tourism options with the objectives of having contact with nature, adopting healthy habits, and understanding introspectively the relationship with the natural environment, among other behaviours produced after the pandemic.

## 6. Conclusions

The aim of this article was to carry out a bibliometric analysis and a literature review of the scientific production on tourism and mushrooms. This analysis has allowed us to provide an overview of the existing documents in this line of research and to analyse the topics related to the economic benefit, welfare, sustainable development, and promotion of the activity. Research into these dimensions increases the conceptual advancement of mycological tourism and the associated scientific knowledge, which will contribute to the foundation and understanding of the link between the bio-cultural resource of mushrooms as socio-economic and welfare factors, as well as the impacts generated through the development of good exploitation practises. As this is a growing topic, we have developed a future research agenda that will guide new research, face the new challenges of the activity, and stimulate reflection. We would like to stress the importance of transferring the knowledge generated to stakeholders. On the one hand, to guide actions that seek to develop sustainable mycological tourism, to associate the scientific interdisciplinarity of research, and to strongly consider innovation and the use of technology in the performance of mycotourism. On the other hand, to design educational content for tourists that will enable them to understand the usefulness and presence of fungi in ecosystems, as well as to know and identify the mushrooms collected, making the tourist a promoter of the care and conservation of this valuable resource.

## Figures and Tables

**Table 1:** Articles. Own elaboration.

Authors	Article Title	Year	Source	Times Cited
García-Nieto, A.P.; García-Llorente, M.; Iniesta-Arandia, I.; Martín-López, B. [37]	“Mapping forest ecosystem services: From providing units to beneficiaries”	2013	*Ecosystem Services*	203
Büntgen, U.; Egli, S.; Galván, J.D.; Diez, J.M.; Aldea, J.; Latorre, J.; Martínez-Peña, F. [38]	“Drought-induced changes in the phenology, productivity and diversity of Spanish fungi”	2015	*Fungal Ecology*	38
Büntgen, U.; Latorre, J.; Egli, S.; Martínez-Peña, F. [1]	“Socio-economic, scientific, and political benefits of mycotourism”	2017	*Ecosphere*	34
Fuste-Forne, F. [39]	“Seasonality in food tourism: wild foods in peripheral areas”	2022	*Tourism Geographies*	18
Liu, D.Y.; Cheng, H.; Bussmann, R.W.; Guo, Z.Y.; Liu, B.; Long, C.L. [21]	“An ethnobotanical survey of edible fungi in Chuxiong City, Yunnan, China”	2018	*Journal of Ethnobiology and Ethnomedicine*	16
De Frutos-Madrazo, P.; Martínez-Peña, F.M.; Esteban, S. [40]	“Edible wild mushroom tourism as a source of income and employment in rural areas. The case of Castilla y Leon”	2012	*Forest Systems*	12
De Frutos, P.; Rodriguez-Prado, B.; Latorre, J.; Martinez-Peña, F. [41]	“A Gravity Model to Explain Flows of Wild Edible Mushroom Picking. A Panel Data Analysis”	2019	*Ecological economics*	9
Latorre, J.; De-Magistris, T.; De Frutos, P.; García, B.; Martínez-Peña, F. [42]	“Demand for mycotourism products in rural forest areas. A choice model approach”	2021	*Tourism Recreation Research*	3
Latorre, J.; De Frutos, P.; De-Magistris, T.; Martínez-Peña, F. [43]	“Segmenting tourists by their motivation for an innovative tourism product: mycotourism”	2021	*Journal of Ecotourism*	2
Rovira, M.; Garay, L.; Górriz-Mifsud, E.; Bonet, J.A. [44]	“Territorial Marketing Based on Non-Wood Forest Products (NWFPs) to Enhance Sustainable Tourism in Rural Areas: A Literature Review”	2022	*Forests*	1
Ovies, D.H.; Bautista, J.J.R. [45]	“Commodified spirituality: tourism and indigenous heritage practices in Huautla de Jimenez, Mexico”	2021	*Tourism Culture & Communication*	0
